# Transmission Characteristics and Predictive Model for Recent Epidemic Waves of COVID-19 Associated With OMICRON Variant in Major Cities in China

**DOI:** 10.3389/ijph.2022.1605177

**Published:** 2022-11-03

**Authors:** Yangcheng Zheng, Yunpeng Wang

**Affiliations:** ^1^ State Key Laboratory of Organic Geochemistry, Guangzhou Institute of Geochemistry, Chinese Academy of Sciences, Guangzhou, China; ^2^ University of Chinese Academy of Sciences, Beijing, China

**Keywords:** COVID-19, China, SEIR model, Omicron, predicting the end of the epidemic

## Abstract

**Objectives:** Waves of epidemics associated with Omicron variant of Coronavirus Disease 2019 (COVID-19) in major cities in China this year have been controlled. It is of great importance to study the transmission characteristics of these cases to support further interventions.

**Methods:** We simulate the transmission trajectory and analyze the intervention influences of waves associated with Omicron variant in major cities in China using the Suspected-Exposed-Infectious-Removed (SEIR) model. In addition, we propose a model using a function between the maximum daily infections and the duration of the epidemic, calibrated with data from Chinese cities.

**Results:** An infection period of 5 days and basic reproduction number R_0_ between 2 and 8.72 are most appropriate for most cases in China. Control measures show a significant impact on reducing R_0_, and the earlier control measures are implemented, the shorter the epidemic will last. Our proposed model performs well in predicting the duration of the epidemic with an average error of 2.49 days.

**Conclusion:** Our results show great potential in epidemic model simulation and predicting the end date of the Omicron epidemic effectively and efficiently.

## Introduction

On 24 November 2021, the World Health Organization (WHO) confirmed a new variant of the 2019 Coronavirus (COVID-19) reported by South Africa which was named as Omicron [1]. WHO rated the global risk of Omicron as “very high,” which could lead to a global pandemic [2]. Last winter, Omicron swept across the world, with the maximum number of daily infections exceeding 3.8 million, and a significant increasing rate of Omicron variant in confirmed COVID-19 positive cases was found [3, 4]. Compared to other variants of the SARS-CoV-2 virus, the Omicron variant has very rapid transmission rate that is much more difficult to predict [1–3].

Since 2022, epidemics of varying severity caused by the Omicron variant have occurred in some major cities in China, with Shanghai and Hong Kong experiencing the most severe outbreaks. Since the Omicron outbreaks, China has continued to insist on the dynamic zero policy of COVID and strategies that have generally proven effective [5]. As of the writing of this article, most cities have succeeded in achieving the goal of dynamic zero COVID. With sporadic relapses occurring in other cities in China, there is no indication that the epidemic will disappear in a short time. Therefore, it is of great importance to comprehensively evaluate and study the current Omicron outbreaks in China to draw lessons for further epidemic control measures.

In this paper, we simulate the course of transmission and the influences of interventions using the Suspected-Exposed-Infectious-Removed (SEIR) epidemic model and analyze the epidemiology and statistical characteristics. In addition, we propose a model using a concise function between the maximum daily infections and the duration of each outbreak, which can be used to predict the end date of ongoing and potential outbreaks in other cities or countries.

## Methods

Twelve typical cases in major cities or provinces in China that have experienced the Omicron epidemic since 2022 were selected as study cases and their daily infection data before 30 April were collected [6, 7]. Since asymptomatic cases are also infectious, we combined the symptomatic cases and asymptomatic cases into daily infections [8, 9]. We mark the date when the number of cases increases and exceeds 1% of the maximum value as the epidemic start date, and the date when the number of cases falls to less than 1% as the epidemic end date. The duration of each outbreak round is defined as the interval between the start and end dates. According to our definition, most selected cases, except Shanghai, experienced one completed outbreak before 30 April, while Shenzhen and Tianjin experienced two completed rounds. The specific start and end dates of each outbreak are shown in Table 1.

**TABLE 1:** Selected cases with Omicron outbreaks (China 2022).

City/province	Start date	End date
Changchun	March 8	30 April
Jilin	March 5	17 April
Hangzhou	January 25	2 February
Shanghai	March 17	N/A
Guangzhou	April 7	22 April
Hong Kong	February 4	16 April
Shenzhen#1^a^	February 19	6 March
Shenzhen#2^a^	March 8	30 March
Tianjin#1^a^	March 8	19 March
Tianjin#2^a^	March 18	14 April
Harbin	April 13	29 April
Shaanxi	March 7	23 March

^a^ #1 and #2 denote the first and second round of epidemic, respectively.

The SEIR model is the most commonly used dynamic epidemic model in COVID-19 studies, which divides the population into suspected population (*S*), exposed population (*E*), infectious population (*I*), and removed population (*R*), whose relationship can be formulated by the following differential equations:

$$\frac{dS\left(t\right)}{dt}=-\frac{\gamma R_{0}\left(t\right)}{N}S\left(t\right)I\left(t\right)$$ (1)

$$\frac{dE\left(t\right)}{dt}=\frac{\gamma R_{0}\left(t\right)}{N}S\left(t\right)I\left(t\right)-\alpha E\left(t\right)$$ (2)

$$\frac{dI\left(t\right)}{dt}=\alpha E\left(t\right)-\gamma I\left(t\right)$$ (3)

where *N* is the total population, *γ* ^−1^ is the infectious period, *α* ^−1^ is the incubation period, and the time-varying *R*_*0*_ *(t)* is defined as follows*:*

$$R_{0}\left(t\right)=\left(R_{0}-R_{1}\right)e^{\left(-q\left(t-T_{i}\right)\right)}+R_{1}$$ (4)

with *R*_*0*_ being the initial basic reproduction number (defined as the average number of secondary infections caused by a single infected individual in a fully susceptible population [10]) and *R*_*1*_ being the final basic reproduction number, *q* being a recession coefficient in the exponential function, and *T*_*i*_ being the intervention time when a significant change can be found in $R_{0}\left(t\right)$ [11]. According to previous reports, the incubation period of Omicron variant is 3 days and *α* is set as 0.33 [11]. The infectious period is the average duration from being infectious to losing infectiousness (death, cured or quarantined). However, the infection period may vary among countries because it is influenced by the ability of detection, quarantine and medical condition. In simulation, we try different values of *γ* from 0.1 to 0.5 with 0.01 intervals to select the most appropriate infection period with best fitting performance.

## Results

**FIGURE 1:**
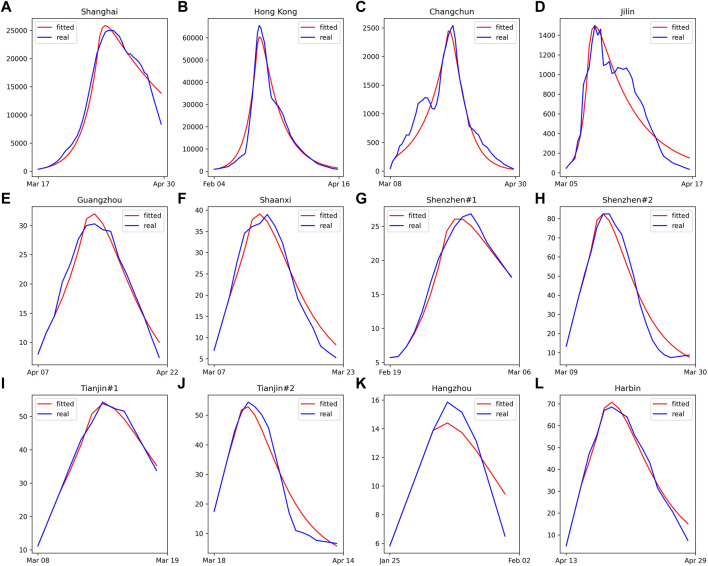
The curves of fitted and actual daily new confirmed cases for the selected cities. **(A)** Shanghai, **(B)** Hong Kong, **(C)** Changchun, **(D)** Jilin, **(E)** Guangzhou, **(F)** Shaanxi, **(G)** Shenzhen round 1, **(H)** Shenzhen round 2, **(I)** Tianjin round 1, **(J)** Tianjin round 2, **(K)** Hangzhou **(L)** Harbin (China 2022).

As shown in Figure 1, our model achieves satisfactory results in most selected cases with *R* ^*2*^ > 0.99. In the simulation, we tried different values of *γ* and found that setting *γ* to 0.2 and corresponding infection duration of 5 days gives the best fitting performance in most selected cases. Under the condition of *γ* = 0.2, the adjusted epidemiological parameters are shown in Table 2. The initial basic reproduction number *R*_*0*_ ranges from 2.01 (Changchun) to 8.72 (Jilin), and after the intervention period *T*_*i*_, $R_{0}\left(t\right)$ decreases to *R*_*1*_, which is less than one in all cases. On average, the date *T*_*max*_ with the maximum daily infections arrives 6 days later than the intervention date *T*_*i*_, and for each day *T*_*i*_ is delayed, *T*_*max*_ is delayed by 2.6 days on average.

**TABLE 2:** Epidemiology parameters of selected cases (China 2022).

City/province	*R*_*0*_	*R*_*1*_	*q*	*T*_*max*_	*T*_*i*_
Changchun	2.01	0.1	3.17	27	31 March
Jilin	8.72	0.51	5.99	10	11 March
Hangzhou	3.56	0	8.95	4	26 January
Shanghai	3.34	0.75	7.76	26	6 April
Guangzhou	3.35	0	5.98	7	11 April
Hong Kong	3.1	0.49	2.28	26	27 February
Shenzhen#1	4.33	0.46	6.4	10	24 February
Shenzhen#2	3.99	0	7.8	7	12 March
Tianjin#1	3.5	0.18	2.21	6	11 March
Tianjin#2	3.03	0	8.79	5	14 April
Harbin	3.92	0	9.15	8	15 April
Shaanxi	3.68	0	4.59	7	10 March

**FIGURE 2:**
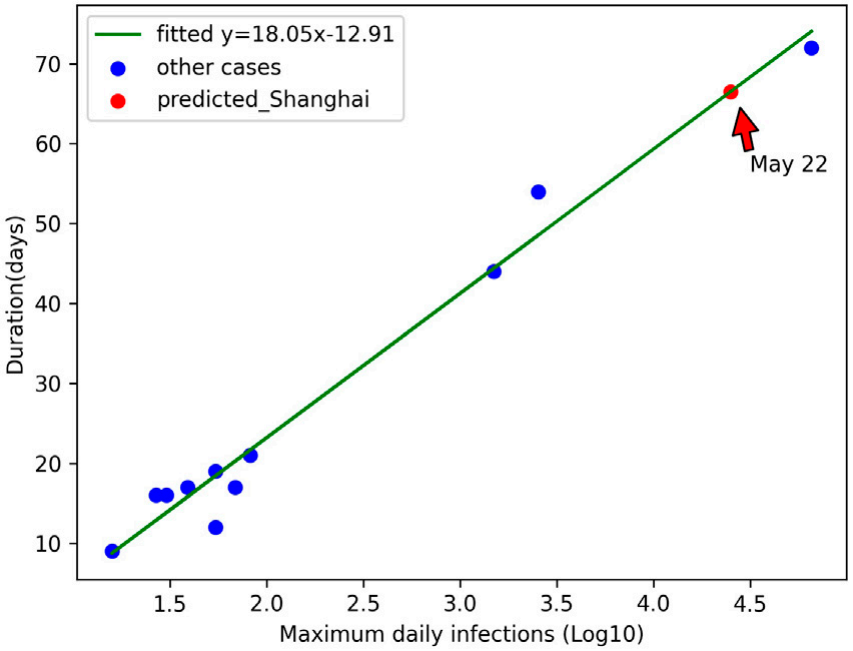
Relationship between the maximum daily infections (Log10) and the duration of the epidemic (China 2022).

We take the logarithm of the maximum daily infections based on 10 and establish its linear relationship with the duration of the epidemic in each selected case (Figure 2). For the cases with completed epidemic, we compare the actual duration with the predicted duration (Table 3), and the error ranges from −5.4 to 6.4 days. According to this function, we predicted that daily infections in Shanghai will decrease to less than 260 (1% of the maximum value) on 22 May, 5 days earlier than the actual date of 27 May.

**TABLE 3:** Selected cases with Omicron outbreaks (China 2022).

City/province	Duration (days)	Predicted duration (days)	Error (days)
Changchun	54	48.6	−5.4
Jilin	44	44.4	0.4
Hangzhou	9	8.8	−0.2
Shanghai	71	66.5	4.5
Guangzhou	16	13.8	−2.2
Hong Kong	72	74	2
Shenzhen#1	16	12.9	−3.1
Shenzhen#2	21	21.7	0.7
Tianjin#1	12	18.4	6.4
Tianjin#2	19	18.4	−0.6
Harbin	17	20.2	3.2
Shaanxi	17	15.8	−1.2

## Discussion

The validity of the SEIR model depends on accurate estimation of the characteristics of virus transmission [12]. A fixed infection rate *γ* = 0.2 may contribute to other studies of Omicron using the SEIR model. The initial basic reproduction number *R*_*0*_ reflects the basic capacity of epidemic prevention and control before the intervention. Except in Jilin city, the *R*_*0*_ of the Omicron variant in selected cases ranges from 2 to 4.4, with an average of 3.43. According to a review of the basic reproduction number for Omicron [13], the average effective *R*_*0*_ is 3.4 with a range of 0.88–9.4, which is highly consistent with our results. The high *R*_*0*_ in the case of Jilin city can be attributed to severe cluster infections in the initial stage [14], but the value decreases significantly after 6 days, and the maximum daily infections come four more days later. Some previous studies have evaluated an extremely high value of *R*_*0*_ of Omicron variant in the very early stage when no control measures were implemented. For instance, *R*_*0*_ could be as high as 10 in the United Kingdom, while the Omicron variant was 3.3 times more transmissible than the Delta variant in the case South Africa [4, 15]. The differences between the values of *R*_*0*_ in the cases of China and other countries may be attributed to the rapid and reasonable reactions to the new sporadic relapses in China [16, 17]. The final reproduction number *R*_*1*_ reflects the transmission ability at which virus spreads in the community after the intervention. In most cases where the dynamic zero COVID target has been reached, *R*_*1*_ is zero or close to zero, indicating that the transmission chain has been completely broken. Our simulation functions, especially Eq. 4, are established under the conditions that effective control measures are implemented and a significant decrease in the transmission rate can be found. In addition, other long-term factors such as seasonality, vaccination, reinfection and demographics are not accounted for in our study for brevity [18].

The control measures implemented in China (i.e., mask wearing and routine nucleic acid testing) are important non-pharmaceutical interventions and can effectively prevent community transmission of the virus [19, 20]. The intervention date *T*_*i*_ is the date when $R_{0}\left(t\right)$ changes appreciably and may not coincide with the date *T*_*max*_, when daily infections reach their maximum and when control measures are implemented. Our results show that the later control measures are taken to break the transmission chain, the more time is spent on controlling the epidemic. Therefore, for other cities adhering to the dynamic zero COVID policy, once new infections are tested, it is important to take control measures as soon as possible to shorten the duration of the epidemic.

We propose a brief linear function to approximately predict the duration of a complete round of epidemics, with error within 1 week. This method is a nonparametric method that is short enough to avoid the uncertainties introduced by manually setting the epidemiological parameters. It should be noted, however, that this method is only applicable to a single epidemic wave and should be used with caution because it is difficult to judge the maximum point.
